# Pain in the lumbar, thoracic or cervical regions: do age and gender matter? A population-based study of 34,902 Danish twins 20–71 years of age

**DOI:** 10.1186/1471-2474-10-39

**Published:** 2009-04-20

**Authors:** Charlotte Leboeuf-Yde, Jan Nielsen, Kirsten O Kyvik, René Fejer, Jan Hartvigsen

**Affiliations:** 1 The Back Research Center, University of Southern Denmark, Ringe, Denmark; 2 Department of Statistics, University of Southern Denmark, Odense, Denmark; 3 Institute of Regional Health Research and The Danish Twin Registry, Epidemiology, Institute of Public Health, University of Southern Denmark, Odense, Denmark; 4 Clinical Locomotion Science, Institute for Sport Science and Clinical Biomechanics, University of Southern Denmark, Odense, Denmark; 5 School of Chiropractic and Sports Science, Murdoch University, Perth, WA, Australia; 6 Nordic Institute of Chiropractic and Clinical Biomechanics, Odense, Denmark

## Abstract

**Background:**

It is unclear to what extent spinal pain varies between genders and in relation to age. It was the purpose of this study to describe the self-reported prevalence of 1) pain ever and pain in the past year in each of the three spinal regions, 2) the duration of such pain over the past year, 3) pain radiating from these areas, and 4) pain in one, two or three areas. In addition, 5) to investigate if spinal pain reporting is affected by gender and 6) to see if it increases gradually with increasing age.

**Method:**

A cross-sectional survey was conducted in 2002 on 34,902 twin individuals, aged 20 to 71 years, representative of the general Danish population. Identical questions on pain were asked for the lumbar, thoracic and cervical regions.

**Results:**

Low back pain was most common, followed by neck pain with thoracic pain being least common. Pain for at least 30 days in the past year was reported by 12%, 10%, and 4%, respectively. The one-yr prevalence estimates of radiating pain were 22% (leg), 16% (arm), and 5% (chest). Pain in one area only last year was reported by 20%, followed by two (13%) and three areas (8%).

Women were always more likely to report pain and they were also more likely to have had pain for longer periods. Lumbar and cervical pain peaked somewhat around the middle years but the curves were flatter for thoracic pain. Similar patterns were noted for radiating pain. Older people did not have pain in a larger number of areas but their pain lasted longer.

**Conclusion:**

Pain reported for and from the lumbar and cervical spines was found to be relatively common whereas pain in the thoracic spine and pain radiating into the chest was much less common. Women were, generally, more likely to report pain than men. The prevalence estimates changed surprisingly little over age and were certainly not more common in the oldest groups, although the pain was reported as more long-lasting in the older group.

## Background

Although there are numerous publications on the prevalence of various types of spinal pain, it would be difficult to compare the relative importance of low back pain (LBP), mid back pain (MBP) and neck pain (NP) through a systematic critical literature review. One of the reasons for this is that it is uncommon for researchers systematically to study the spine from a multiple-area approach. In addition, it is particularly rare to find epidemiologic data on the thoracic spine.

Other reasons making it difficult to compare results between publications are that studies differ in their selection criteria of study populations, methods of data collection, definitions of the anatomical sites, cut-off points for pain reporting, and prevalence periods. Further, the thoracic spine, if at all included, is usually not defined to include the entire T1 – T12 area. Instead, either part of the thoracic area may be covered or it is combined with, for example, the shoulder region. Comparisons might be further confused by variations occurring over time and between geographical regions and cultures. All this is likely to result in incomparable study results, as has been shown previously [1-3].

The results of a non-exhaustive search of the epidemiologic back pain literature of the Nordic populations indicate that also the influence of gender is uncertain. Although in many studies, LBP, MBP or NP were more commonly reported in women than in men [4-11], this was not always the case in other studies or in the same studies when different definitions of pain were used [4,7,12,13].

The same confusion exists in relation to age. Sometimes LBP, MBP or NP was noted to increase with age [6,12,13]. In other cases it was found to peak in the middle years [4,5,8,13], and in yet others to remain the same across all ages or to diminish with age [4,9].

In order to gain more insight into this area, identical data were collected for each of the three spinal regions and studied in relation to gender and age in a population-based study of almost 35,000 twin individuals aged 20 to 71. To provide maximum information on age, it was presented as a continuous variable. In addition, we reported on pain in one, two or all three regions, and, finally, on the prevalence of pain radiating from these three spinal regions, i.e. pain into the leg, the chest, or the arm.

## Methods

### Study design, data collection and validity of data

This was a cross-sectional study of the Danish general population using twin individuals. The subjects were recruited from The Danish Twin Registry, which is over 50 years old and holds data on more than 75,000 twin pairs born from 1870 to 2004. The completeness is between 27% and 73% before 1968 and almost 100% for those born after 1968 [14]. In 2002, all twins born between 1931 and 1982, who had previously consented to take part in research, were sent a 20-page questionnaire including questions from many different research groups. The information letter stated the purpose of the projects as focusing on twins' health in general. The questionnaire was followed by one reminder, which was the number of reminders allowed by the Danish Scientific Ethical Committees at that time. The study had the required permissions from the regional Scientific Ethical Committees and Data Protection Agency (file number: 20010201).

In population-based studies of twin individuals it has been shown that twins report health-related findings corresponding to those found in the general population, such as the prevalence of diabetes [15,16] and can therefore be used with confidence in epidemiologic studies. In the present study, preliminary analyses of the data revealed that there were no significant differences in the prevalence of back pain between the two zygotic groups, indicating that there was no accumulation of findings among the identical twins. Also the mortality is the same as in the general population [17]. Further, the present twin cohort was found to be similar to the general Danish population on age, civil status, working status, and type of residence. There were, however, small discrepancies in relation to gender (more women in the twin cohort), level of income (higher), rate of retirement (fewer twins were retired), and education (twins were more likely to have a longer education) [18].

No wave-analysis was undertaken to attempt to extrapolate the profile of the non-responders. However, a comparison between responders and non-responders in our study revealed that non-responders were more likely to be males, younger, single, divorced/widowed, retired, unemployed, self-employed, or to have lower income [19].

### Variables of interest

A translation of the spinal pain questionnaire is provided in Additional File 1. In relation to LBP, MBP and NP, respectively, the following information was collected: Pain ever, pain in the past year, number of days with pain in the past year (later divided into 1–7 d, 8–30 d, >30 d). Also pain reported as radiating from the lumbar, thoracic, or cervical areas was included, described as pain in the leg(s), chest, and arm(s), respectively. All variables except the "ever" variables relate to symptoms in the past year.

### Analysis and presentation of data

Not all study subjects answered all the relevant questions. When missing data could easily be corrected by logical imputation, this was done. An example of this is a person, who answered "yes" to having had LBP in the past year but did not respond to the question on LBP ever, which would result in a corrected "yes" for LBP ever. Another example is a person who failed to report LBP in the past year, although he reported some consequence of LBP in the past year (such as having sought care because of LBP), which would be classified as having had LBP in the past year. This procedure resulted in less than 1% missing pain data.

Descriptive data are shown for the whole study sample, mainly as graphs. In addition, the data are presented by age, and separately for men and women. Because numerous estimates are provided in this report, the text will be explanatory mainly, i.e. relating to similarities and differences, peaks and slopes, rather than providing exact estimates. Continuous data are shown surrounded by their 95% point wise confidence intervals. If estimates are surrounded by clearly separated point wise confidence intervals, they will be considered to be statistically significantly different. When such separation between intervals is sporadic, it will be described as "sometimes significantly different". Differences between estimates will be noted also if their point wise intervals overlap providing that these differences are consistent.

The consequences of back pain, the effect of other covariates than age and gender, and the genetic aspect will be reported elsewhere.

## Results

### Description of the study sample

**Figure 1:**
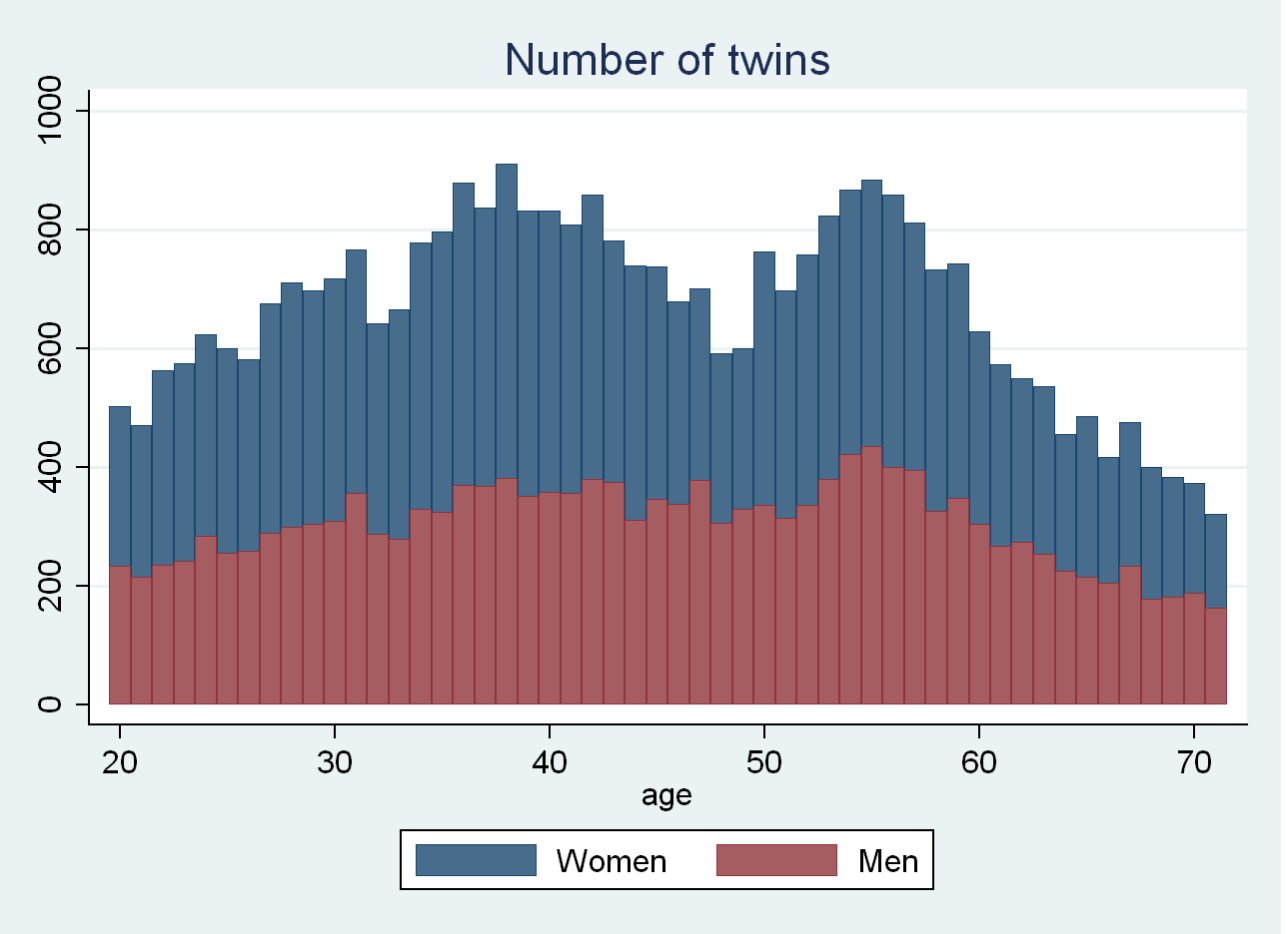
**The number of male and female individuals who participated in a Danish omnibus survey by age (n = 34,902)**.

A total of 34,902 valid questionnaires out of 46,818 (74%) were returned, 19,877 in the first round and the rest after one reminder. Of the valid questionnaires, 54.5% were provided by females. In total, 30% were aged 20–35, 33% were 36–50 yrs, 37% were 51–71 years of age, and 68% were work-active. The 38-year olds made up the largest age group (n = 918) and the 71-year olds the smallest (n = 164). The number of responders for each age group was proportional to the number of potential responders in the targeted study sample. The number of respondents was less than 400 for each of the three oldest groups (69–71 yrs), which resulted in less stable estimates and larger confidence intervals, especially when dividing the sample into men and women (Fig. 1)

### The prevalence of self-reported spinal pain for the whole study sample

Sixty-nine percent reported to have had some sort of spinal pain in the past and 55% reported that it had happened in the past year. LBP was most frequently reported, followed by NP. MBP was much less common (Table 1).

**Table 1:** The prevalence estimates of different definitions of spinal pain in a study of 34,902 Danish twin individuals.

**Definition of variables**	N (%)*
**LBP **ever	20,053 (57%)

LBP past year	15,093 (43%)**

LBP 1–7 days	3,804 (10%)

LBP 8–30 days	6,168 (18%)

LBP >30 days	4,207 (12%)

Pain radiating into leg(s)	7,651 (22%)

**MBP **ever	5,966 (17%)

MBP past year	4,535 (13%) **

MBP 1–7 days	1,161 (3%)

MBP 8–30 days	1,633 (5%)

MBP >30 days	1,338 (4%)

Pain radiating into chest	1,846 (5%)

**NP **ever	14,059 (40%)

NP past year	11,316 (32%) **

NP 1–7 days	2,523 (7%)

NP 8–30 days	4,345 (12%)

NP >30 days	3,641 (10%)

Pain radiating into arm(s)	5,583 (16%)

* Because of the large sample size, confidence intervals would be very narrow (usually 1 unit), and have therefore not been included in the table.

** The number of days for 1–7 days, 8–30 days and >30 days do not add up to the year-estimates, because the latter have been corrected, if data were missing, based on subsequent answers in relation to site-specific consequences in the past year.

Regardless of the spinal area, the most commonly reported total period of pain in the past year was 8–30 days, followed by more than 30 days, and then 1–7 days (Table 1).

Of the radiating symptoms during the past year, leg pain was most common, followed by arm pain and chest pain (Table 1).

It was most common that respondents recalled having had pain in one spinal area only (usually LBP), followed by two and three areas (20%, 13%, and 8%, respectively). For the 13%, who reported to have had pain in two areas, this was usually in the lumbar and cervical spine (10%).

### The prevalence of self-reported LBP, MBP or NP in relation to age and gender

**Figure 2:**
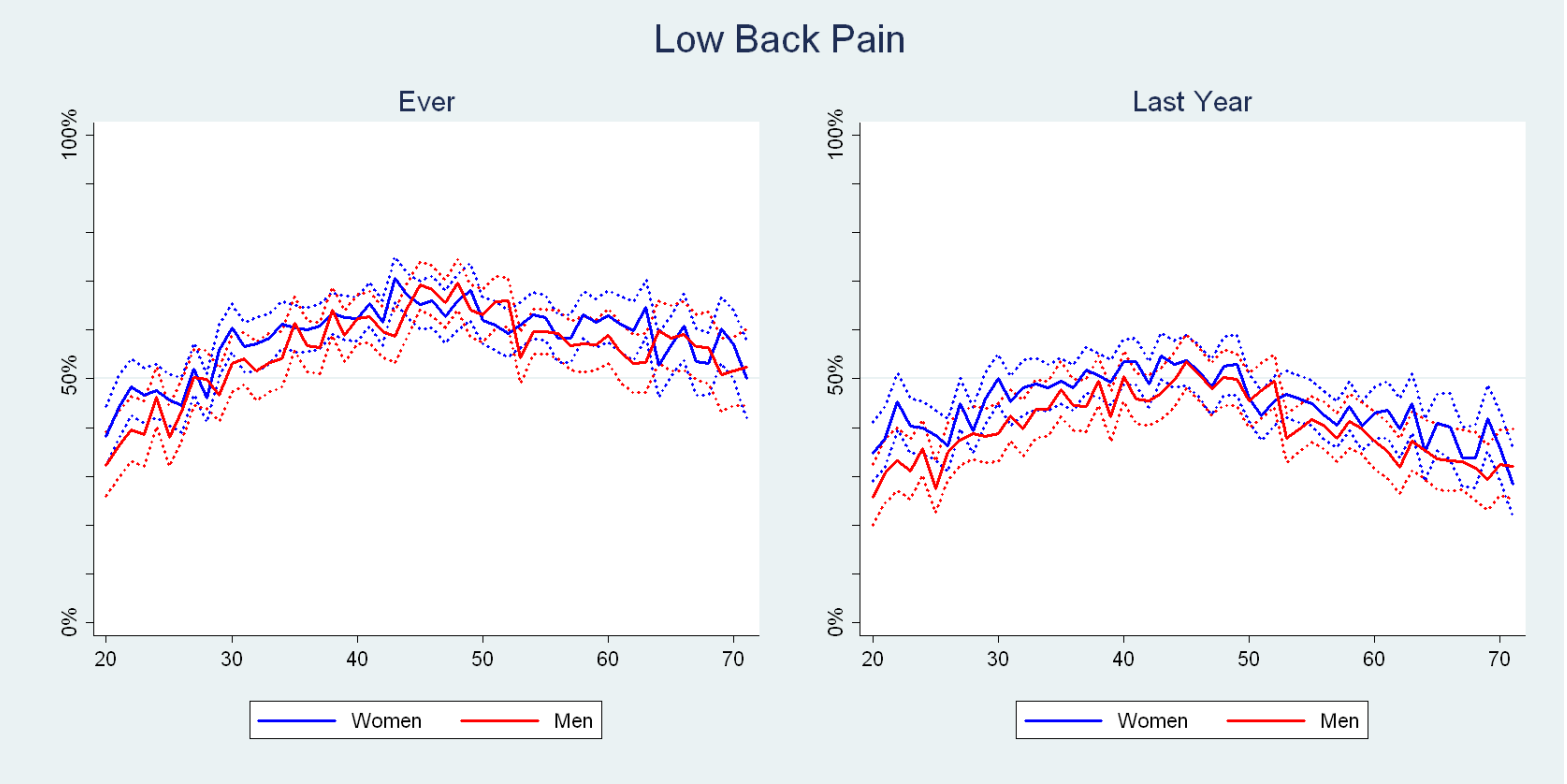
**The proportions of Danish twin individuals aged 20 to 71 who reported to have had low back pain ever and those who reported to have had low back pain in the past year by age and gender (N = 34,674)**.

The reporting of LBP ever and LBP in the past year peaked around the age of 45 for both men and women, with a 1-yr period prevalence estimate of about 55% for both genders. Although, generally, the estimates for women were higher than the estimates for men, the point wise confidence intervals overlapped (Fig. 2).

**Figure 3:**
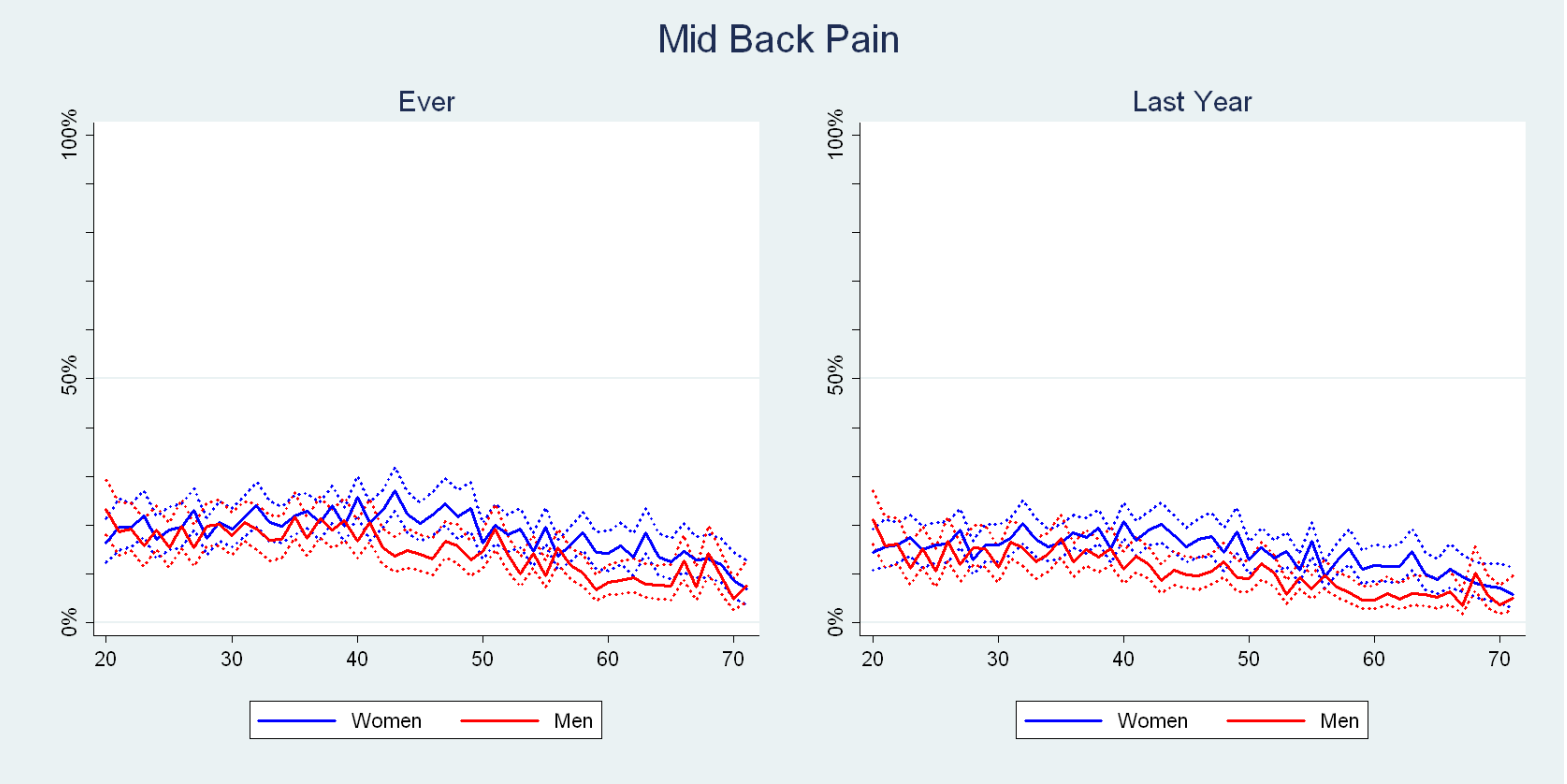
**The proportions of Danish twin individuals aged 20 to 71 who reported to have had mid back pain ever and those who reported to have had mid back pain in the past year by age and gender (N = 34,674)**.

The distributions of MBP ever and MBP in the past year were seen as two relatively flat curves, which both descended somewhat from the age of 40 for both men and women. Women were consistently more likely to report MBP than men and after the age of 40 estimates were significantly different (Fig. 3). At the age of 40, for example, 20% of the women had MBP in the past year but only 10% of the men.

**Figure 4:**
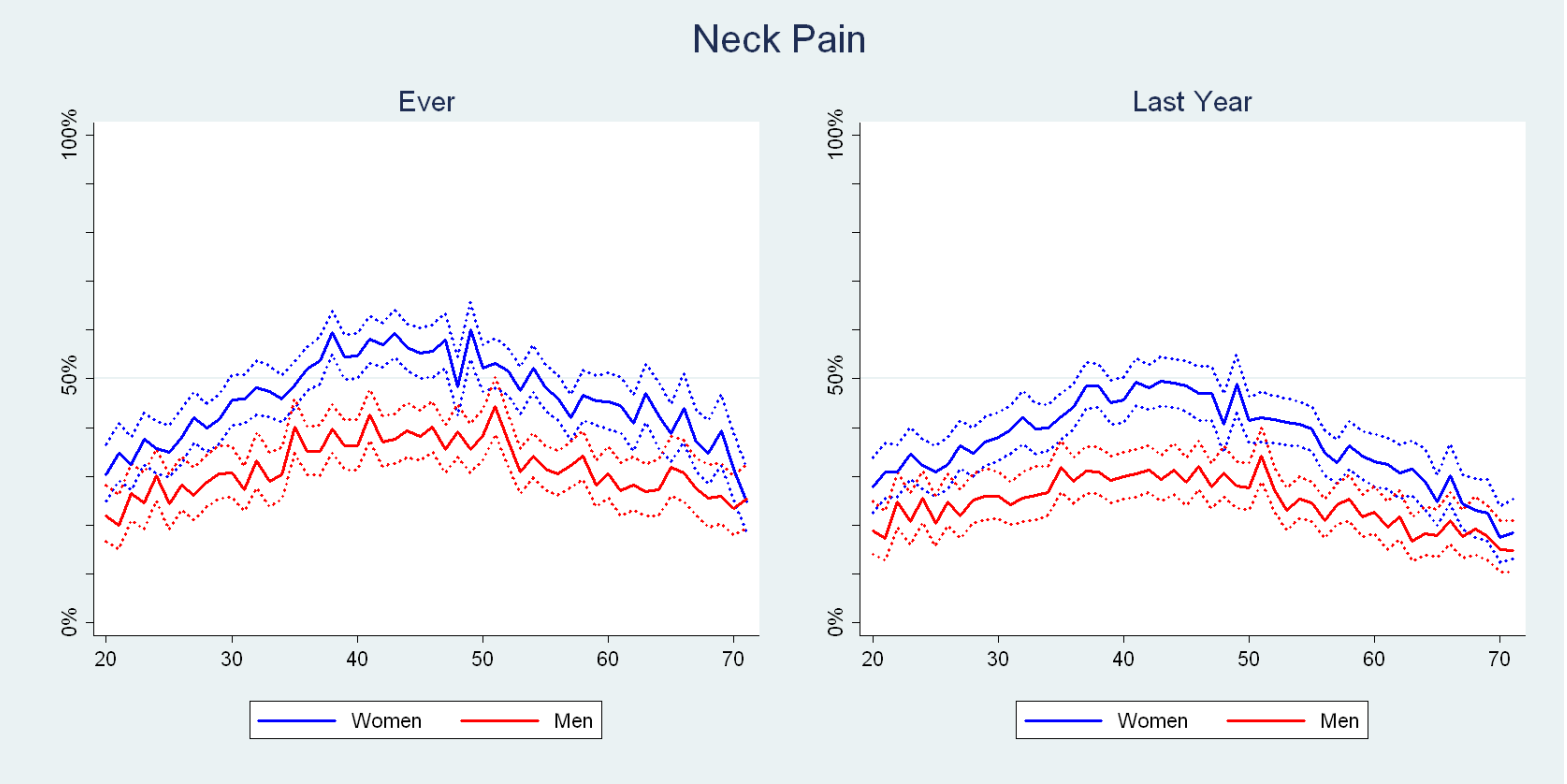
**The proportions of Danish twin individuals aged 20 to 71 who reported to have had neck pain ever and those who reported to have had neck pain in the past year by age and gender (N = 34,674)**.

NP ever and NP in the past year peaked between 40 and 50, to descend at the age of 70 towards or below the same level as at the age of 20. The estimates were significantly different with the higher estimates consistently noted for women (Fig. 4). At 40, the 1-yr period prevalence was about 45% for women but only 30% for men.

**Figure 5:**
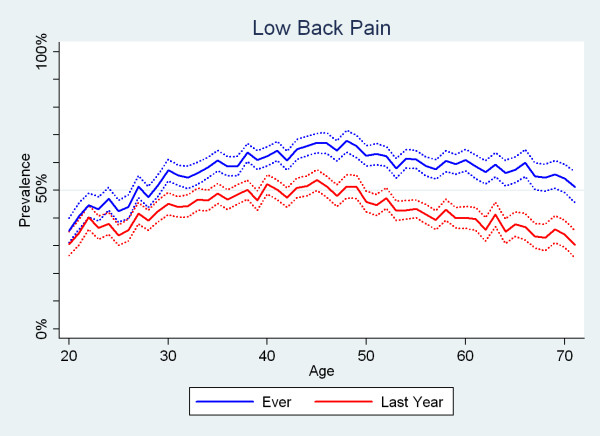
**The proportions of Danish twin individuals aged 20 to 71 who reported to have had low back pain ever and those who reported to have had low back pain in the past year by age (N = 34,674)**. The zigzag patterns of the two curves are very similar.

In addition, it was noted that the curves of the prevalence estimates for the past year and ever were almost parallel for all three spinal regions over the various age-groups and both genders. The curves for LBP are shown as an example (Fig. 5).

The remaining results will be reported only in relation to pain in the past year.

### The number of days with pain in the three spinal regions during the past year

**Figure 6:**
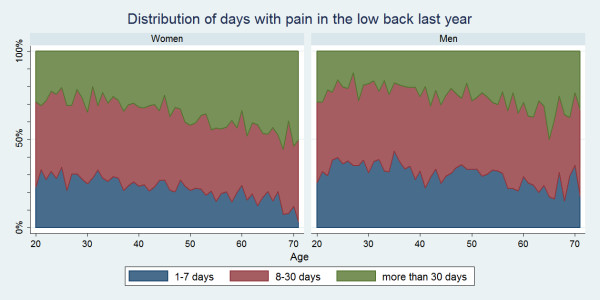
**Graph showing the number of days in the past year that Danish twin individuals aged 20 to 71 had low back pain among those who did have low back pain in the past year**. Data are shown separately for men and women (N = 15,093).

**Figure 7:**
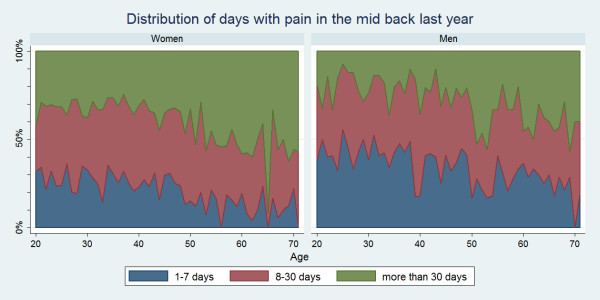
**Graph showing the number of days in the past year that Danish twin individuals aged 20 to 71 had mid back pain among those who did have mid back pain in the past year**. Data are shown separately for men and women (N = 4,535).

**Figure 8:**
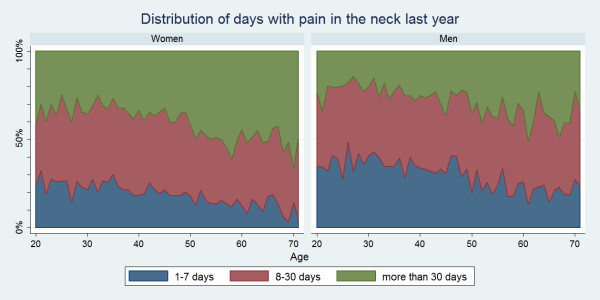
**Graph showing the number of days in the past year that Danish twin individuals aged 20 to 71 had neck pain among those who did have neck pain in the past year**. Data are shown separately for men and women (N = 11,316).

There were positive associations between age and number of days with pain for all three regions. Women were more likely than men to report LBP, MBP or NP for altogether >30 days in the past year and across the age groups this was sometimes significantly different for LBP and NP (data not shown). At the other extreme, LBP, MBP or NP for altogether 1–7 days in the past year was more commonly reported for men, and across the age groups this was sometimes significantly different for LBP and NP (data not shown). The duration of pain is illustrated in Figs. 6, 7, 8.

### The 1-yr period prevalence of self-reported pain radiating into the legs, chest and arms

**Figure 9:**
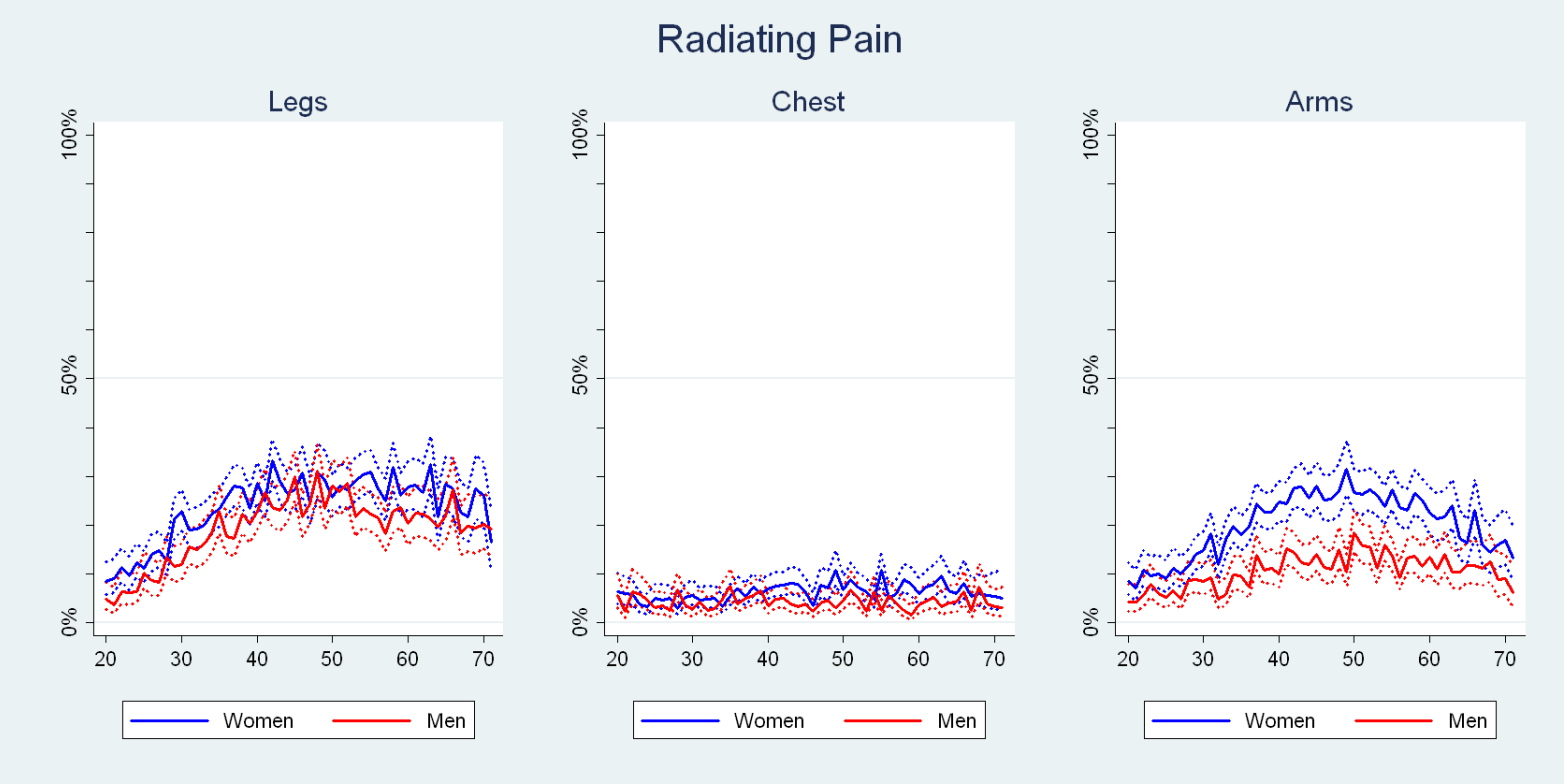
**The proportions of Danish twin individuals aged 20 to 71 who reported to have had radiating into the leg, chest or arm in the past year**. The data are shown separately for men and women (N = 18,993).

The curves for radiating pain by age and gender are shown in Fig. 9. Radiating leg pain increased markedly up to the early 40s to reach about 30%, whereupon the curve flattened out. Women were more likely than men to report leg pain, and between the ages of 30 and 40, this was sometimes significantly different.

Radiating chest pain exhibited a fairly flat curve in the vicinity of 5%. The estimates were for the most part higher for the women than for the men. Between the late 40s and the late 60s, these gaps were sometimes significantly different.

Radiating arm pain peaked around the age of 50. The prevalence was consistently higher for women than for men and between the ages of 30 and the early 60s this was significantly different. At the peak, the prevalence was 30% for the women and almost 20% for the men.

### The 1-yr period prevalence of self-reported spinal pain in one, two or three areas in relation to age and gender

**Figure 10:**
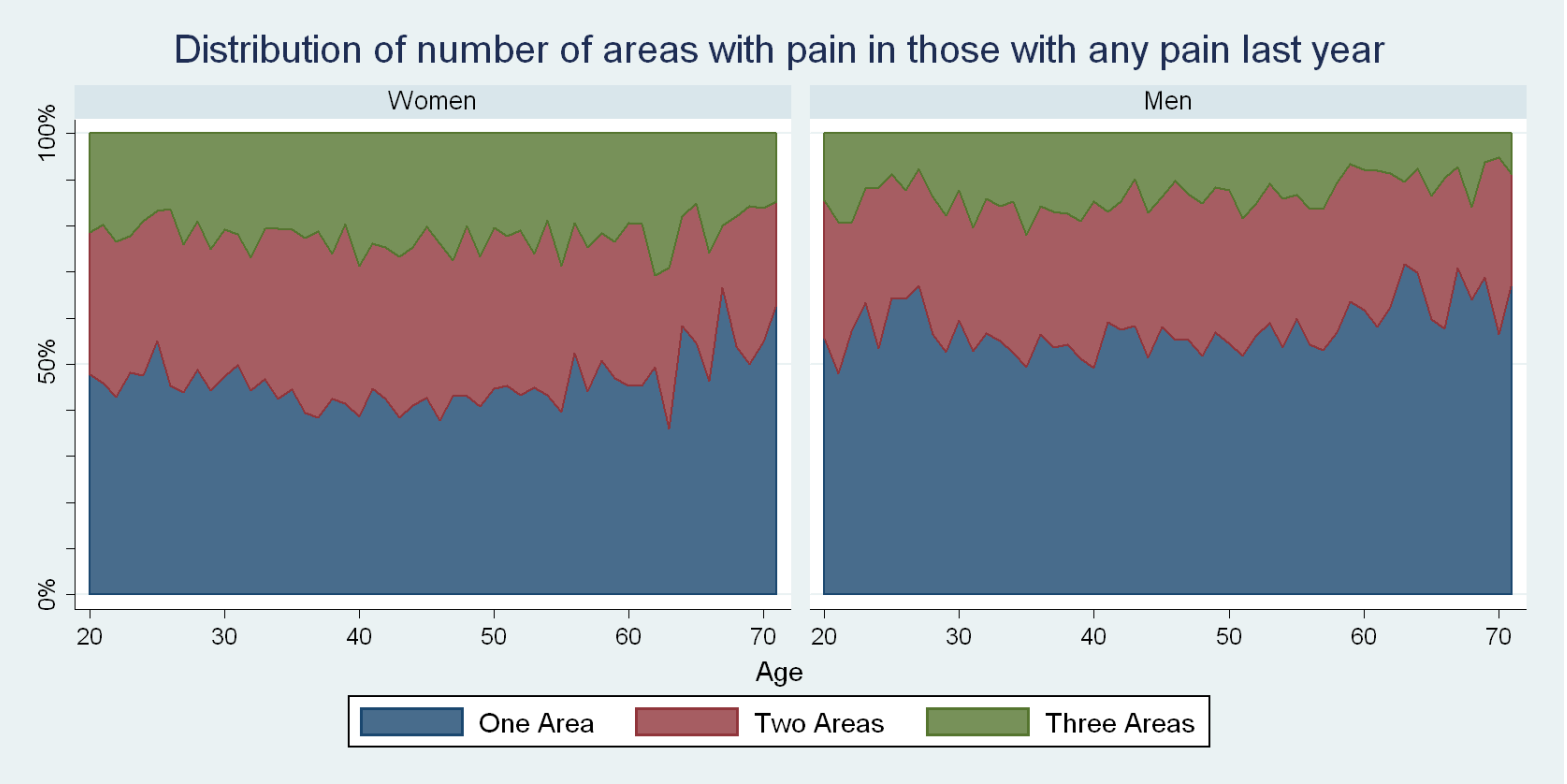
**The proportions of Danish twin individuals aged 20 to 71 who reported to have had pain in one, two or all three areas of the spine among those with spinal pain in the past year**. The data are shown separately for men and women (N = 34,902).

The number of painful spinal areas did not increase gradually with age (Fig. 10). Women were more likely to report pain in two or three areas, and for three areas this was often significantly different (data not shown).

### In summary

#### In general

• At least 55% of the study sample reported to have had pain in at least one spinal area in the past year.

• Fifty percent of those with spinal pain in the past year, reported pain in one area only, and this was the most common finding.

• Spinal pain in the past year was most commonly reported as LBP (43%), closely followed by NP (32%) and, far behind, by MBP (13%).

• The radiating pain pattern had a similar anatomical hierarchy, with leg pain being most common (22%), followed by arm pain (16%), and, rarely, chest pain (4%).

• The "ever" and "past year" curves had very similar profiles for all 3 spinal regions.

• In the past year, pain for 8–30 days was most common, followed by pain for >30 days, whereas pain for 1–7 days was least common.

#### In relation to age

• Pain in more than one area peaked at the ages of 35–45.

• In relation to pain ever and pain in the past year, the curves of LBP and NP resembled each other in that they peaked around the middle years, whereas the curves for MBP were flatter.

• The curves for radiating pain were similar to those for the area from which the pain radiated.

• There was a positive association between age and number of days with pain in the past year, regardless of the area of pain, and the increase was gradual.

#### In relation to gender

• Pain in more than one region was more common in women than in men.

• Women as compared to men were more likely to report spinal pain, regardless of the area of the spine, and the difference was most marked for NP.

• Women were also more likely than men to report radiating pain, regardless the area.

• Women were more likely than men to have had pain for 8 days or more.

## Discussion

### Some surprising findings

This study offered some surprising results. The finding that intrigued us the most was that the oldest group did **not **report pain in more areas than the younger groups. This indicates that spinal pain does not accumulate with age. An explanation could be that if there is an inherited or acquired tendency for spinal pain in an individual, it is likely to manifest itself early in life, but if there is no such weakness, it will not occur, regardless of what happens in life. Thus the simple fact of getting older will not result in an increased burden of spinal pain.

Another remarkable finding was the relatively even pattern of pain reporting across the ages regardless of the area of pain. With the exception of MBP, pain reporting was already common at the age of 20, increasing slowly until the middle years to descend slowly again. This mid-life peak phenomenon has been noted by others for various parts of the spine [4,5,8]. That the pain reporting is more pronounced at that time in life might be the result of a poor balance between the abilities of the spine and the demands of daily living. The downward slope that follows in the oldest group has been previously noted, the potential reasons for which have been extensively discussed by Helme [20].

It also surprised us that the duration of pain was reported similarly regardless of the area of pain; a total duration of 8–30 days was most commonly reported, followed by >30 days. This pattern has been described before in a previous study of LBP in Danish twins, at that time aged 12–41-yrs [19], but it has not been previously shown that this is also the case for pain in the thoracic and cervical spines.

The fourth surprise was that the occurrence of radiating pain resembled the pattern of the area from which the pain probably originated. In other words, radiating leg pain was more common than radiating arm pain, with radiating chest pain being uncommon. The clinical relevance of these findings merits some reflections. It could indicate that the causes of radicular pain are intrinsic (common anatomical factors or genetics) rather than related to extrinsic factors such as environment and life-style.

This study resulted in some new information, namely that pain in and from the thoracic spine, although relatively rare, has a pattern that is fairly similar to that of the neck and low back. This should be of particular interest for clinicians, as the pain in the thoracic spine often raises concerns about a spinal pathology. However, "non-specific mid back pain", although less common may have a similar (as yet largely unknown) etiology and course as non-specific low back or non-specific neck pain. In other words, also for mid back pain, the proportion of spinal pathologies may in fact be very low.

### Some expected findings

Not surprisingly, this study revealed that LBP is commonly reported, followed by NP whereas MBP is relatively rare in the general Danish population. We were able to identify only two studies from the Nordic countries, in which all three specific parts of the spine were studied, with findings reported separately for men and women across the ages, and where no additional areas, such as shoulders or chest, were included in the definitions of neck and thoracic spine. In one of these studies (including 2,726 20–72-yr old Norwegians), the same pattern of prevalence as in our study was found but for men only [4]. This was the case also in the other study, in which 1,422 18–58-yr old Swedes were studied [7].

Also as expected, for all spinal regions, women were most often afflicted, both when reporting on number of painful areas and on the number of days with pain. The reasons why women report more back pain than men are unknown but the finding is not surprising, as this is in line with the increased prevalence of illness reporting among women in general [21].

Although the older groups did not report pain in a larger number of areas of the spine, there were, nevertheless, signs of increased persistence of back problems with increasing age relating to all three parts of the spine, indicating that acute pain does depend on a physiological repair process that slows down with age.

### Present and future methodological considerations

This appears to be the first study of the general population, in which it was clearly reported on the prevalence of LBP, MBP and NP across such a wide range of age groups in both men and women.

Other strong points of our study are that the study sample is likely to be well representative of the general Danish population and sufficiently large to ensure precision of estimates across most age groups.

The response rate is good compared to many other surveys, and the study sample is unlikely to be biased as it did not consist mainly of people interested in back pain. The questions on back pain were accompanied by drawings that clearly showed the anatomical areas of interest. There were few missing answers throughout the questionnaire indicating that the questionnaire was user-friendly. Nevertheless, the pain-profiles of the non-responders are unknown and they may well affect the external validity of our data, as is usually the case in studies of this type.

Although the numbers of our smallest age groups (the oldest participants) more than equalled the numbers of many subgroups in smaller epidemiologic studies, the zigzag pattern of the prevalence curves increased markedly towards the older age groups, which consisted of fewer study subjects. Also the confidence intervals widened considerably. Obviously, even in studies with representative study samples, already for subgroups of about 400 there is a risk for imprecise estimates. Therefore, one should be careful when interpreting results from studies that are based on small study samples or small subgroups, such as when reporting on different age groups, particularly when their findings deviate from the "usual" findings.

In future research, some other methodological precautions should be taken into consideration. For example, the pattern of the pain reporting "ever" for each age group was remarkably similar to that of pain in the past year in all three spinal regions. This could reflect an extreme recurrence of back pain but it could also indicate that the long-term memory is highly dependent on the short-term memory and that the "ever" variable is fairly useless.

Another point to consider is that there are some different age-related patterns of back pain reporting for different definitions of pain and that there is no linear increase. This makes age-adjustment of estimates rather tricky. The practice of grouping study subjects into age groups is problematic also, since unsuitable cut off points may conceal patterns that might be seen with continuous data reporting, and furthermore, according to our data, different pain variables require somewhat different cut off points.

## Conclusion

In conclusion, pain in and radiating from the lumbar and cervical spines was relatively common, whereas pain in and radiating from the thoracic spine was much less common. When spinal pain was present, the findings in relation to duration, radiating pain, and age- and gender-related findings were remarkably similar for the three spinal regions. Women were, generally, more likely to report pain than men. Although there were differences in pain reporting between the different age groups, there is no general and consistent trend for a gradual increase with age with the exception of the duration of pain, which becomes increasingly more apparent with increasing age. Contrary to our expectations, the older groups were not more likely than the younger groups to report pain in more than one area.

## Competing interests

The authors declare that they have no competing interests.

## Authors' contributions

KOK was responsible for the entire twin project; CLY, JH and RF secured the funding; CLY and JH designed the back pain questionnaire; JN did the statistical analyses and designed the illustrations. CLY formulated the research questions, interpreted the data and wrote the first draft. The whole group commented on the manuscript and all authors read an approved the final manuscript.

## Pre-publication history

The pre-publication history for this paper can be accessed here:



## Supplementary Material

Additional file 1 **Questionnaire**. English translation of the questions on back pain used in the Danish omnibus study. Click here for file
